# Effectiveness of Social Cognitive Theory–Based Interventions for Glycemic Control in Adults With Type 2 Diabetes Mellitus: Protocol for a Systematic Review and Meta-Analysis

**DOI:** 10.2196/17148

**Published:** 2020-09-02

**Authors:** Yvonne Smith, Rosalia Garcia-Torres, Steven S Coughlin, Jiying Ling, Terri Marin, Shaoyong Su, Lufei Young

**Affiliations:** 1 College of Nursing Augusta University Augusta, GA United States; 2 Family and Consumer Sciences California State University Northridge, CA United States; 3 Department of Population Health Sciences Augusta University Augusta, GA United States; 4 College of Nursing Michigan State University East Lansing, MI United States; 5 Georgia Prevention Institute Augusta University Augusta, GA United States

**Keywords:** social cognitive theory, type 2 diabetes mellitus, glycemic control, self-efficacy, self-management, HbA1c, glycosylated hemoglobin

## Abstract

**Background:**

For those living with type 2 diabetes mellitus (T2DM), failing to engage in self-management behaviors leads to poor glycemic control. Social cognitive theory (SCT) has been shown to improve health behaviors by altering cognitive processes and increasing an individual’s belief in their ability to accomplish a task.

**Objective:**

We aim to present a protocol for a systematic review and meta-analysis to systematically identify, evaluate, and analyze the effect of SCT-based interventions to improve glycemic control in adults with T2DM.

**Methods:**

This protocol follows the 2009 Preferred Reporting Items for Systematic Review and Meta-Analysis (PRISMA) guidelines. Data sources will include PubMed, Cumulative Index to Nursing and Allied Health Literature (CINAHL), PsychINFO, Cochrane Library, and Web of Science, and data will be reviewed with the use of customized text mining software. Studies examining SCT-based behavioral interventions for adults diagnosed with T2DM in randomized controlled trials located in the outpatient setting will be included. Intervention effectiveness will be compared with routine care. Screening and data collection will be performed in multiple stages with three reviewers as follows: (1) an independent review of titles/abstracts, (2) a full review, and (3) data collection with alternating teams of two reviewers for disputes to be resolved by a third reviewer. Study quality and risk of bias will be assessed by three reviewers using the Cochrane risk of bias tool. Standardized mean differences will be used to describe the intervention effect sizes with regard to self-efficacy and diabetes knowledge. The raw mean difference of HbA1c will be provided in a random effects model and presented in a forest plot. The expected limitations of this study are incomplete data, the need to contact authors, and analysis of various types of glycemic control measures accurately within the same data set.

**Results:**

This protocol was granted institutional review board exemption on October 7, 2019. PROSPERO registration (ID: CRD42020147105) was received on April 28, 2020. The review began on April 29, 2020. The results of the review will be disseminated through conference presentations, peer-reviewed journals, and meetings.

**Conclusions:**

This systematic review will appraise the effectiveness of SCT-based interventions for adults diagnosed with T2DM and provide the most effective interventions for improving health behaviors in these patients.

**Trial Registration:**

PROSPERO CRD42020147105; https://www.crd.york.ac.uk/prospero/display_record.php?RecordID=147105

**International Registered Report Identifier (IRRID):**

PRR1-10.2196/17148

## Introduction

### Background

It is estimated that 9.4% of the American population is living with diabetes mellitus, and of these, 90%-95% are diagnosed with type 2 diabetes mellitus (T2DM) [1]. Insulin resistance characterizes T2DM and is often precipitated by lifestyle-associated risk factors [2]. Managing T2DM requires the application of a large amount of knowledge to maintain consistent behaviors. Other requirements for effective diabetes self-management are high levels of discipline and diligence for prudent decision making. Complex medical regimens and poor perception of an ability to control the disease worsen self-management adherence [3,4]. The inadequacy of diabetes self-management and suboptimal glycemic control have been well documented in a national prevalence study involving over 4900 participants. The study examined the prevalence of people who met glycemic targets from 1988 to 2010. Of the 4900 participants, only 2572 (52.5%) experienced glycemic control with a HbA_1c_ value of less than 7% and 2313 (47.2%) of participants had a HbA_1c_ value of over 7% [5,6]. Deficient glycemic control has been attributed to individual pathophysiology progression, poor self-management skills, lack of support, and nonadherence to a healthy lifestyle [5]. The Center for Disease Control and Prevention describes the severe complications of poor glycemic control. Complications include cardiovascular disease, cerebrovascular disease, limb amputation, retinopathy, and renal failure [2].

### Theoretical Behavioral Interventions

The use of theory in behavioral research is not new. Theory-based behavioral interventions have been shown to produce longer and lasting glycemic control in adults with T2DM [7,8]. Behavioral interventions direct behavioral change through cognitive pathways [9]. Individualized theoretically based behavioral interventions have been shown to be more effective than a generalized curriculum or routine care alone [8,10,11].

In a scoping literature review examining how current research presents theory-based interventional studies, several goals were considered as follows: (1) which theory or theories were used, (2) which concepts were used as the foundation of an intervention, and (3) what variables were measured compared to the theory and concepts that were identified (Y Smith, MSN, et al., unpublished data, 2019). Among the three criteria, there was one similarity and some inconsistencies that were noted. The one similarity was that one theory consistently held favor among the scientific community and was more commonly used than any other theory. This theory was social cognitive theory (SCT). Despite collective judgment pointing to the effectiveness of SCT, there are variations of its use within the current literature. For example, (1) only a single concept was identified as the focus, (2) some studies identified one concept but measured another instead, (3) findings were inconsistently reported as both effective and ineffective, and (4) numbers one, two, and three combined were seen in some studies. At initial glance, the inconsistencies could lead to a false conclusion that there is no prevailing effective or singularly dominant theory to reduce glycemia. A deeper investigation is warranted of the use and the reported effectiveness of SCT-based trials. Neglecting further inspection would disregard an apparent consensus on SCT within the scientific community. A systematic review and meta-analysis on the most prevalent theory would confirm or deny the current scientific consensus and identify the most effective interventions to reduce glycemia in adults with T2DM.

### Social Cognitive Theory

SCT aims to promote self-management behavior (eg, adopting a healthy lifestyle) through self-regulating cognitive processes [12]. Cognitive processes are more than receiving an education or performing a skill; they are defined as managing complex health conditions (eg, T2DM) to obtain a desired response (eg, optimal glycemic control) [13-15]. Self-management behavior is the cornerstone of effective diabetes care. Based on the SCT, cognitive processes promote self-management behavior by improving knowledge, self-efficacy, and problem-solving skills [16].

**Figure 1:**
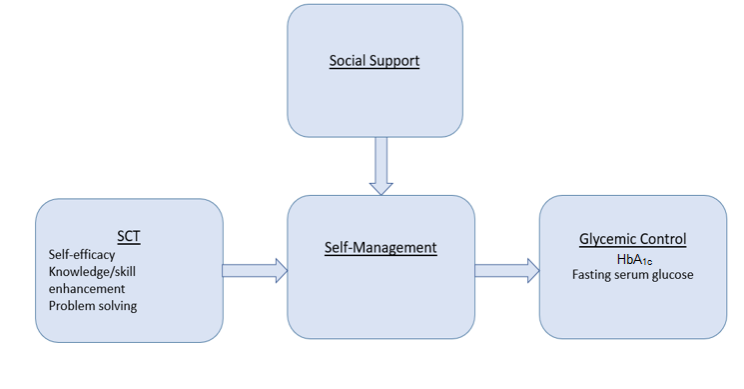
Social cognitive theory–based interventions for glycemic control. SCT: social cognitive theory.

Emerging from the scoping review is the SCT-based framework (Figure 1), which has guided this research. In this framework, glycemic control is the outcome of self-management behavior. It is defined as a HbA_1c_ value of less than 7%, fasting serum glucose level between 70 and 130 mg/dL, and serum glucose level of 180 mg/dL 2 hours after a meal [16]. Self-management behavior is made up of various complex daily activities required to reach glycemic targets [4,17-20] and defined as self-monitoring serum glucose, healthy eating, regular physical activity, stress management, problem solving, medication adherence, and goal setting [21-23]. First, SCT-based interventions promote self-management behavior by enhancing diabetes self-management knowledge, which is defined as the general knowledge of diabetes self-management [23]. Second, SCT-based interventions improve self-efficacy, which is defined as the belief one has in themselves to perform a behavior [12]. Lastly, SCT-based interventions improve self-management by enhancing problem-solving skills defined as a series of cognitive operations used to figure out what to do when the way to reach a goal is not apparent [17,24,25]. Social support moderates self-management and includes informational support by providing education or advice. Instrumental support can be financial support or physical assistance with self-management actions. Emotional support provides acceptance and approval, whereas affirmational support provides validation of self-management efforts [17,26,27].

### Text Mining

A methodological challenge in conducting this systematic review is the presence of an overwhelming amount of information on adults with T2DM. A GALILEO search using SCT and T2DM as search terms revealed well over 100,000 results. Search results from a variety of databases will include full-text studies, abstracts, books, conference materials, and grey literature, which are discussed in the search strategy below.

To better explore the immense amount of textual evidence, this review proposes to extract information by using the method of text mining [28]. Text mining is a technology-based application that uses semantics as a method of discovery [29]. Although text mining is widely adopted in other fields, there are currently no detectable systematic reviews on theory-based interventional research using this innovative technique. Another strength of text mining is semantic annotation that allows relationships, keywords, or topics to be attached to the concept [30]. The rationale for using this innovative method is exploration to (1) accurately manage the massive amount of literature found for prompt screening, (2) compare text-mined results to reviewer results, (3) show the numerical frequency of the identified concepts in the included literature, (4) know the strength and position of a concept thereby gaining insight into possible mechanisms of action, and (5) create a visual word cloud of the results [31]. The use of text mining enhances the discovery of knowledge and will improve the quality of data extraction. By identifying how the concept is involved in the text, assertions can be made, and they ultimately will increase the fidelity and results of this review.

### Objectives

The purpose of this review is to examine the effect of SCT-based behavioral interventions to improve glycemic control in adults with T2DM. We initially hypothesize that patients who receive an SCT-based intervention have better glycemic control. We further hypothesize that patients who receive greater social support have better glycemic control.

The research goals are as follows:

1. To examine the relationships between glycemic control and concepts (ie, self-efficacy, knowledge, and problem-solving) targeted by SCT-based behavioral interventions in adults living with T2DM.
2. To examine the pooled effect of SCT-based interventions on glycemic control (ie, HbA
  1c
  and fasting serum glucose) in adults living with T2DM.
3. To examine the interaction between social support and SCT-based interventions for glycemic control in adults living with T2DM.

## Methods

### Design

**Figure 2:**
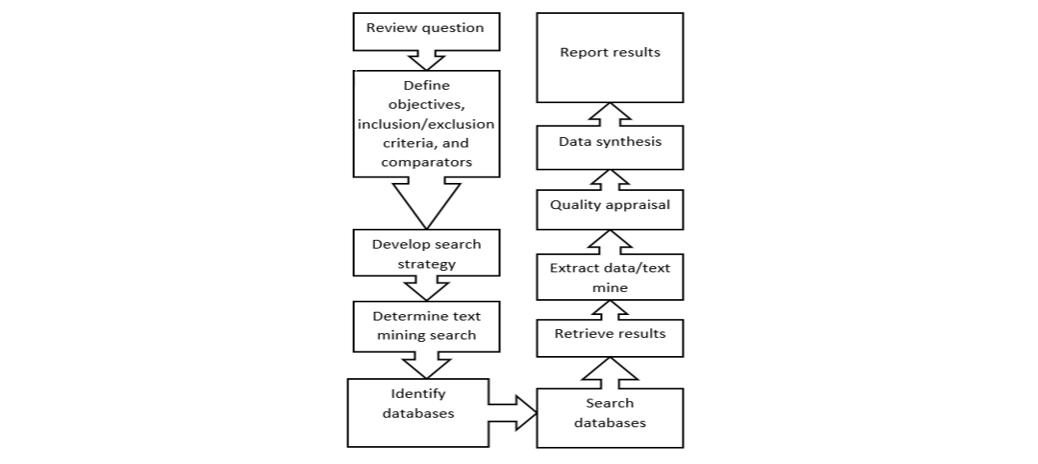
Stages of the systematic review.

This systematic review follows the 2009 Preferred Reporting Items for Systematic Review and Meta-Analysis (PRISMA) guidelines. The stages of the systematic review are presented in Figure 2.

This systematic review will examine randomized controlled trials owing to their inherent strength. Studies with a less rigorous methodology, such as nonrandomized trials, quasiexperimental or observational studies, research protocols, and drug trials, will be excluded. No date restrictions will be applied in searching for eligible studies.

### Eligibility

The inclusion criteria are presented in Textbox 1. Studies targeting health care professionals; studies including individuals diagnosed with type 1 diabetes mellitus (T1DM), gestational diabetes, prediabetes, drug-induced diabetes, or metabolic syndrome; and studies including individuals aged less than 18 years will be excluded. Studies set only in the outpatient setting will be included. There will be no restrictions on gender, socioeconomic status, ethnicity, or race.

**Inclusion criteria.**

**Inclusion criteria**

Population: Outpatient setting, previously diagnosed with type 2 diabetes mellitus, and age over 18 years

Intervention: Interventions based on social cognitive theory (SCT) concepts or interventions that use a combination of SCT and another theory, model, or framework, with a minimum 3-month time frame

Comparison: Routine care

Outcomes: HbA_1c_ and fasting serum glucose

Study design: Experimental designs including randomized controlled trials

### Outcomes

The primary outcome of interest is glycemic control. Glycemic control will be assessed before and after the intervention. All glycemic control outcomes will be observed and recorded objectively.

#### Glycemic Control

The HbA_1c_ test is a blood test measuring the average blood glucose level over the past 3 months. Glucose adheres to hemoglobin located within red blood cells. The adherence of glucose to red blood cells is referred to as glycosylated hemoglobin or HbA_1c_ [1]. The secondary measures of glycemic control are fasting blood glucose and postprandial blood glucose.

All studies lasting less than 3 months will be excluded, as HbA_1c_ takes 3 months to manifest. There will be no exclusions regarding the length of follow up. Studies with no comparison, an additional comparison, or an alternative comparison will be excluded, as it is inappropriate to provide anything less than routine care. Included concepts of SCT are self-efficacy, skills, practice, motivation, self-regulation, attitude, expectations, knowledge enhancement/acquisition, skill enhancement/acquisition, social norms, social network, social support, community, experience mastery, efficacy expectation, problem-solving, verbal persuasion, vicarious experience, physiological feedback, reflection, and reward.

### Search Strategy

As mentioned above, a GALILEO search using SCT and T2DM search terms presents thousands of results. Completing a GALILEO search begins with the development of key terms; however, this alone can produce results that cover other topics. A comprehensive search strategy has been developed and will be used to complete the literature search in four stages by three reviewers.

Keywords and phrases are derived from the research goals and are the foundation of the search strategy. The search will be completed in four stages. Stage one will involve the development of search terms phrases, descriptions, attributes, or sentences for determining text mining parameters [30]. Examples of key SCT concepts and derived strategies are presented in Table 1. Key concepts and search terms have been gleaned from a previous scoping literature review completed between 2016 and 2018 (Y Smith, MSN, et al., unpublished data, 2019).

**Table 1:** Social cognitive theory concepts and derived strategies.

Theory	SCT^a^ concepts	Derived strategies
SCT	Social learning, social cognitive, feedback, knowledge, attitude, self-efficacy, self-efficacy, confidence, problem-solving, problem solving, coping, coping strategy, vicarious, verbal, motivational, self-regulation, attitudinal, belief, mastery, efficacy expectation, accomplishment, verbal persuasion, vicarious experience, physiological or affective state, physiology feedback, social norms, social network, social support, community, experience mastery, efficacy expectation, and reward	Soap opera, guided group discussion, group cooking, cooking demonstrations, group meals, presentations, messages, self-monitoring demonstrations, cognitive reframing, quizzes, modeling, family support, behavioral “experiments” (or trials), stress management, label reading, review log, reinforcement of positive attitudes, visual aids, goal setting, group “games,” support, planning, literacy, color-coded graph, traffic light, log, track, personalized counseling, identifying-strategy, role play or role-play, reflection, group counseling, encouraging, positive feedback, demonstrating, role modeling, assessment, follow-up, follow up, worksheet, and self-help

^a^SCT: social cognitive theory.

Stage two will begin with a broad search of keywords and phrases. An example of a beginning search string is presented in Multimedia Appendix 1. An initial broad search will be used to identify relevant studies and to assist in expanding keywords and phrases for a more in-depth search. A previous search for any systematic reviews on this topic only revealed one study on theory-based educational interventions for adults with T2DM [8]. However, currently, there is no systematic review meta-analysis on SCT-based behavioral interventions for adults with T2DM. To our knowledge, there is no review using text mining in the review process. Existing systematic reviews will be assessed to expand keywords and phrases for a more in-depth search. At minimum, Medline, Cumulative Index to Nursing and Allied Health Literature (CINAHL), and PubMed will be searched during stage two.

Stage three will be an expanded search of key concepts, terms, strategies, and phrases and again will include Medline, CINAHL, and PubMed, along with Web of Science, Ovid, PsycINFO, and Cochrane Library. Additionally, the approach will include ProQuest, a dissertation database, grey literature, a hand search, and a search of the reference lists of included studies. The Boolean operators “AND” and “OR” will be used as connectors for keywords, and mesh terms will be used. Additionally, professional organizations relevant to the review will be searched for reports, guidelines, and unpublished research (eg, the American Diabetes Association).

Stage four will involve a search of the reference lists of identified articles for any relevant references, systematic reviews, and meta-analyses, and a hand search of appropriate journals. Additionally, an author search will be conducted for any publications by Zhao et al [8], as this is currently the only known similar systematic review.

There will be no date restrictions on the searches. Database searches will include peer-reviewed journals, and full-text studies only will be included with reported results. No language restriction will be applied; however, if a study is not translatable, it will be excluded. As the search strategy progresses, a detailed record of search activities will be kept (Multimedia Appendix 2). The timeframe for searching each database will also be maintained; further, if the entire database since inception is not used, a valid reason will be provided. As eligible citations are identified, they will be organized in the EndNote X7 citation manager and then downloaded by the first reviewer into an online publication screening manager (Rayyan) for title and abstract screening.

### Publication Selection Criteria

Reviewers will work independently initially and then in teams of two to allow the third reviewer to settle any disputes that may arise. The review team will include a doctoral student as the first reviewer, a doctorally prepared dietician researcher as the second reviewer, a doctorally prepared nursing researcher and a major advisor as the third reviewer, a reference librarian, and a statistician. The first and second reviewers have completed training specializing in systematic reviews and meta-analyses.

**Figure 3:**
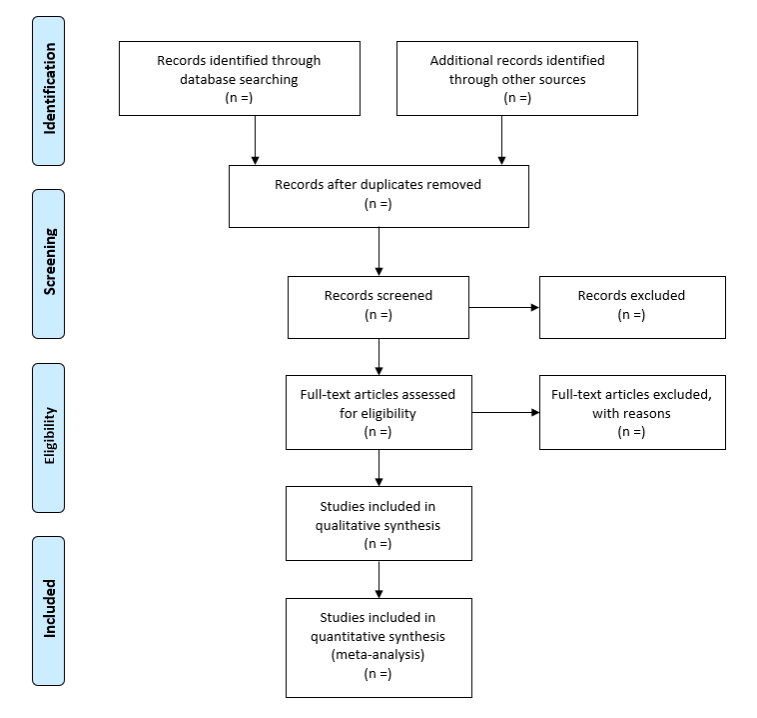
Flow diagram.

A record of search results will be compiled into numbered lists by the first reviewer to create the search log (Multimedia Appendix 2) and will be used to develop the study flow diagram (Figure 3). The search log will document each database search and all rationale for excluding any titles, abstracts, or studies by the first reviewer. Entries for the search log will include the number of included and excluded studies, along with the rationale for exclusion from the independent and full-text review.

The initial screening will be completed by three reviewers on Rayyan, which enables reviewers to screen blindly and independently. The first screening will be of titles and abstracts; duplicates will be removed and noted in the search log. Reviewers will then meet to discuss and take decisions on titles and abstracts that are conflicting in the independent review. Studies that meet the inclusion criteria will be organized in a separate folder in a shared file on Dropbox, and the full-text articles will be retrieved by the first reviewer. Full-text screening will be performed in teams of two reviewers, and data will be collected in the codebook (Multimedia Appendix 3). A hand search will be completed, and included publications will be added for the full-text review.

Text mining will be completed by a project collaborator proficient with customized text mining software. Full-text publications will be preprogrammed and screened by word recognition (Multimedia Appendix 4). Results will be compiled into an excel file (Multimedia Appendix 5). Reviewers will use the text frequency table, systematic annotations, and word cloud to compare results from screening, data extraction, and analysis.

### Quality Assessment

The review team will follow the 2009 PRISMA checklist to evaluate included studies (Multimedia Appendix 6). Further, the unit of analysis will be assessed to determine at which point randomization occurred to ensure participants are not duplicated in the results. To account for missing data, we will critically evaluate the number of participants before the intervention versus after the intervention, the attrition rates, and if an intention-to-treat analysis was completed. If missing data for calculating effect sizes are present, corresponding authors will be contacted for further information. If two unsuccessful attempts are made to reach a corresponding author within 2 weeks, the article will be excluded. Assessment of the quality of individual studies will be completed by examining the risk of bias and will be conducted while coding. The Cochrane collaboration tool for assessing risk of bias will serve as the guide for assessing bias in the included literature. Judgments for selection, performance, detection, attrition, reporting, and selection bias will be based on answering “yes” or “no” to specific criteria outlined in Multimedia Appendix 7. The two reviewers will assess the risk of bias according to the requirements above and discuss any disputes with the third reviewer.

### Data Extraction and Management

The review team will conduct data extraction and management in an Excel spreadsheet. The first reviewer will work closely with the reference librarian to produce an effective search string of all literature. The first and second reviewers and major advisor understand research design and have the ability to critique research studies. The first and second reviewers will code independently and settle any disputes with the third reviewer. A statistician will ensure that proper analyses are conducted for the different types of data extracted. Extracted data will be coded and defined according to the codebook example in Multimedia Appendix 3. The codebook will be the foundation for a table presenting the characteristics of the included studies. The characteristics of the included studies and their extracted data are reported in an Excel table (Multimedia Appendix 8).

### Data Analysis

Standardized mean differences will be used to describe the intervention effect sizes with regard to self-efficacy and diabetes knowledge. To calculate the standardized mean differences, the means, standard deviations, and change scores of the self-efficacy and knowledge evaluation scales will be recorded [13,23-25,32-35].

The standardized mean difference will be considered small (value of 0.2), moderate (value of 0.5), or large (value of 0.8). Individual and overall effect sizes, 95% CIs, variance, and *P* values will be reported. For measures of glycemic control, including HbA_1c_, finger stick blood sugar, postprandial serum glucose, and random serum glucose, the weighted mean differences will be calculated as the intervention effect sizes. The weighted mean will preferably be calculated with pre-HbA_1c_ levels, change scores, and *P* values, if available. The effect size, variance, and direction of the effect will be evaluated and compared. Individual and overall analyses will be presented in a forest plot and described in the narrative [36].

Heterogeneity will be assessed by visual inspection of the forest plot and chi-square (*Q*) with a significance level of .05, and compared using the *I^2^* statistic. The *Q* statistic and *P* value offer statistical evidence of heterogeneity. The *I^2^* details the ratio of between-study variance to total variance and is a display of the magnitude of heterogeneity. The range of *I^2^* is 0 to 100, and an *I^2^* value of >50% will be considered to indicate significant heterogeneity. If heterogeneity is indeed present, we will perform a meta-regression to identify where the variation may lie and explain the variation in the narrative. The meta-regression will be performed to explain covariates or characteristics at the study level. A random-effects model will be used [36].

When heterogeneity is present, a subgroup analysis will be conducted to determine if there is a difference between studies that reported SCT exclusively and studies that reported SCT and the use of another theory, model, or framework. The research aims to determine which variation of theory (SCT alone or SCT combined with another theory) is more effective in the analysis. A subgroup analysis will be conducted on those studies reporting a self-efficacy component of the intervention versus a knowledge or skill-enhancing component. Additionally, the research aims to determine if the duration of T2DM has any impact on effect size. Subgroup analysis will be performed by analyzing a pooled or separate *T*^2^ depending on the number of studies in each subgroup and the within-study variance [36]. Additionally, a subgroup analysis will be performed on the moderator of support to determine if any difference exists between the treatment group and the routine care group.

The funnel plot will be examined to assess publication bias by performing a Begg and Mazumdar rank correlation and an Egger regression test. The Begg and Mazumdar test and Egger test determine if the effect size is reflective of the study sample size. The funnel plot will be analyzed to note the presence of symmetry by assessing the distribution of individual effect sizes surrounding the *x*-axis versus the standard error on the *y*-axis. To evaluate whether the overall effect is an object of bias, Orwin fail-safe *N* will be used. Orwin fail-safe *N* allows the determination of how many missing studies are needed to detect an effect greater than zero. Additionally, Orwin fail-safe *N* will be used because it does not require the adjusted effect size to be zero [36].

Additionally, the impact the bias has on the overall effect will be assessed by the Duval and Tweedie trim and fill procedure. The Duval and Tweedie procedure identifies the studies causing an asymmetrical funnel plot. The procedure recalculates the overall effect size of bias to determine how much impact bias has on the overall effect size and if there is a change in the effect when bias is adjusted. Language bias will be assessed by comparing the effect size between English studies and non-English studies, if necessary. The impact of English and non-English studies will be determined by observing the overall effect when English studies are removed from the analysis. Confounding variable bias will be assessed with a subgroup analysis to know whether (1) SCT alone was used versus SCT and another theory, (2) the duration of T2DM changes the effect, or (3) the intervention components impact the overall effect. Judgments regarding the risk of bias (low, moderate, or high) will be made by the reviewing team based on Cochrane criteria for judging the risk of bias [36].

Data analysis will be completed using comprehensive meta-analysis (CMA) [36]. A summary of the findings table will include a random effect meta-analysis to estimate individual and overall effect sizes. A 95% CI and a *P* value will be reported with effect sizes. Further, a summary discussing study limitations, consistency of effects, imprecision, indirectness, and publication bias will be presented. If substantial heterogeneity exists, subgroup analysis and meta-regression for continuous variables will be performed. A subgroup analysis will be performed on the moderator if supported. A tau-squared statistic will be reported, and an explanation of between-study variance will be provided. A sensitivity analysis will be completed to determine the impact of decisions made during the review, if any. Additionally, a sensitivity analysis will be completed to assess the effect of outliers, if any. It is difficult to name specific items that will be analyzed in this protocol owing to attributes that are unidentifiable until the review and analysis are completed.

### Data Synthesis

The data will be presented in summary of findings tables and will serve as the foundation for discussing the results. Analysis of the raw mean differences will be provided in a random effects model and presented in a forest plot. A discussion of the results of each outcome will be presented in narrative form. Further, a discussion of the results of the subgroups and sensitivity analysis will be presented. For each study, the consistency of the pooled effect, precision or imprecision of the effect, and bias will be evaluated and discussed. The results of the study characteristics are presented in Multimedia Appendix 8. A reflection of the methods specified in this proposal will be provided to present any issues that arose in the discussion section. Finally, a discussion in a narrative format on the clinical significance of the results will be provided.

## Results

This protocol was granted institutional review board exemption on October 7, 2019, by Augusta University in Augusta, Georgia. PROSPERO registration (ID: CRD42020147105) was received on April 28, 2020. The review began on April 29, 2020. As of May 27, 2020, there are 43 publications included in the review. The results of the study will be disseminated through conference presentations, peer-reviewed journals, and meetings.

## Discussion

As with any study, there are expected limitations that will need to be addressed. The limitations include publications reporting incomplete data or analyzing various types of glycemic control measures accurately within the same data set (eg, HbA_1c_ and fasting serum glucose). The authors of studies with missing data will be contacted and given 2 weeks to respond. We will consult with an expert statistician to guide data entry and analysis of the various forms of glycemic measures within the same analysis. Another noted limitation is accurately comparing and interpreting the text-mining results with reviewer results and the CMA results. To our knowledge, there is no other social science study utilizing text mining.

Further, based on our information, this study utilizes the most comprehensive data collection tool of any similar study, with over 90 data points. This protocol is designed based on previous research on the use of various theories to form effective interventions for adults with T2DM. This work will appraise the effectiveness of SCT-based interventions by analyzing the pooled effect of SCT-based interventions on glycemic control. The exploration of reviewer results and text-mining results will produce insights unknown before this study, with the ultimate goal of informing health care providers on the most effective behavioral interventions for improving glycemic control.

## Appendix

Multimedia Appendix 1 Search string example.

Multimedia Appendix 2 Search log.

Multimedia Appendix 3 Codebook.

Multimedia Appendix 4 Social cognitive theory concepts and descriptions.

Multimedia Appendix 5 Excel text mining example.

Multimedia Appendix 6 PRISMA quality checklist.

Multimedia Appendix 7 Cochrane risk of bias tool.

Multimedia Appendix 8 Characteristics of the included studies.

## Abbreviations

CMA: comprehensive meta-analysis
SCT: social cognitive theory
T1DM: type 1 diabetes mellitus
T2DM: type 2 diabetes mellitus
