# Supplementary material for: Effectiveness of Social Cognitive Theory–Based Interventions for Glycemic Control in Adults With Type 2 Diabetes Mellitus: Protocol for a Systematic Review and Meta-Analysis
Source: JMIR Res Protoc. 2020 Sep 2;9(9):e17148. doi: 10.2196/17148 (PMC7495254; doi:10.2196/17148)
Supplement: Multimedia Appendix 1 [file resprot_v9i9e17148_app1.docx]

**#1**

“Type 2 Diabetes” [tiab] OR (“Type 2 Diabetes” OR “T2DM” OR “Adult Onset Diabetes”)

**Results 122,720**

**#2**

"Glycated Hemoglobin A"[Mesh] OR "hemoglobin A1c protein, human" [Supplementary Concept] OR "Blood Glucose"[Mesh] OR “glycemic control” OR “fasting blood glucose” OR “fasting blood sugar” OR A1C [tiab] OR “glycosylated hemoglobin”

**Results 203,321**

**#3**

“Social Cognitive Theory” OR “guided intervention” OR “Bandura's social” OR “social-cognitive”[tiab] OR “Social learning theory”[tiab] OR “Learning theory”[tiab] OR “Social cognitive”[tiab] OR “Self-efficacy” OR "self efficacy" OR “Efficacy theory” OR “Social learning”[tiab] OR “cognitive”[tiab] OR "Self-Management"[Mesh] OR "Reward"[Mesh] OR "Motivation"[Mesh]

**Results 542,868**

**#4**

Clinical Trial [ptyp] OR Controlled Clinical Trial [ptyp] OR systematic [sb]

**Results 993,804**

#1 AND #2 AND #3 AND #4
