# Supplementary material for: Effectiveness of Social Cognitive Theory–Based Interventions for Glycemic Control in Adults With Type 2 Diabetes Mellitus: Protocol for a Systematic Review and Meta-Analysis
Source: JMIR Res Protoc. 2020 Sep 2;9(9):e17148. doi: 10.2196/17148 (PMC7495254; doi:10.2196/17148)
Supplement: Multimedia Appendix 2 [file resprot_v9i9e17148_app2.docx]

| **1** | **2** | **3** | **4** | **5** | **6** | **7** |
| --- | --- | --- | --- | --- | --- | --- |
| **Date** | **Database(s) or search location(s)** | **Keywords or search term used** | **# of Articles Retrieved after** | **# of Articles included after**  **Title and Abstract review** | **# of Articles included after full-text review** | **Total number excluded articles and number of sub-categories with the rationale** |
|  | PubMed | See search string documents |  |  |  | Duplicates:  No theory:  Wrong study design:  Wrong population:  Wrong outcomes:  Study protocols without study results:  Not behavioral intervention study:  Full text unavailable in English:  Full text unavailable:  Unreadable:  **____excluded after title/abstract screening, and ___excluded after full text review, so a total ___ excluded** |
