# Supplementary material for: Effectiveness of Social Cognitive Theory–Based Interventions for Glycemic Control in Adults With Type 2 Diabetes Mellitus: Protocol for a Systematic Review and Meta-Analysis
Source: JMIR Res Protoc. 2020 Sep 2;9(9):e17148. doi: 10.2196/17148 (PMC7495254; doi:10.2196/17148)
Supplement: Multimedia Appendix 3 [file resprot_v9i9e17148_app3.docx]

|  | | | **Variable** | | | | **Explanation** | | | | | |
| --- | --- | --- | --- | --- | --- | --- | --- | --- | --- | --- | --- | --- |
| **Report & Study Information** | | | | | | | | | | | | |
|  | Paper ID | | | | | |  | | | | | |
|  | Year of publication/distribution | | | | | |  | | | | | |
|  | Frist Author name | | | | | |  | | | | | |
|  | Country the study conducted | | | | | |  | | | | | |
|  | Funding  0 = none reported  1 = yes, for-profit group (e.g., drug company)  2 = yes, none profit (e.g., NIH)  3 = yes, funding from for-profit and non-profit sources  4 = yes, unclear if for profit or not for profit funder | | | | | |  | | | | | |
|  | Source  1 = journal article  2 = dissertation/thesis  3 = book/chapter  4 = presentation/abstract of presentation  5 = manuscript/unpublished report | | | | | |  | | | | | |
|  | Title | | | | | |  | | | | | |
|  | Purpose | | | | | |  | | | | | |
|  | Study design | | | | | | RCT, Quasi-experimental | | | | | |
|  | | Were individual subjects randomized to Tx and Co groups?  0= No/not mentioned or not clear in report  1 = yes, randomized by individual subject | | | | | | | | | | |
|  | | Were data collectors blinded/masked to the assignment of subjects?  0 = data collectors were NOT blinded to subject assignment as stated in  report  1 = yes, data collectors were blinded to subject group assignment as stated in  report  2 = blinding/masking of data collectors to subject group assignment was not  clearly discussed in the report. | | | | | | | | | | |
|  | | Study setting  0 = unable to determine/ not applicable  1 = urban/suburban  2 = rural | | | | | | | | | | |
|  | | Type of control  0 = unable to determine/ not applicable  1 = usual or standard care, no additional interventio  2 = attention or partial attention (give intervention that is irrelevant to DM mgt)  3= wait list, will get intervention after study  4 = Usual or standard care + DM related attention | | | | | | | | | | |
|  | Inclusion and Exclusion Criteria | | | | | |  | | | | | |
| **Sample Characteristics:** | | | | | | | | | | | | |
|  | | Mean age intervention group | | | | |  | | | | | |
|  | | Age SD intervention group | | | | |  | | | | | |
|  | | Mean age control group | | | | |  | | | | | |
|  | | Age SD control group | | | | |  | | | | | |
|  | | Percent of the sample that are women in Tx group | | | | |  | | | | | |
|  | | Percent of the sample that are women in control group | | | | |  | | | | | |
|  | | Percent of the sample that are white | | | | |  | | | | | |
|  | | Percent of the sample that are black | | | | |  | | | | | |
|  | | Percent of the sample that are Hispanic | | | | |  | | | | | |
|  | | Percent of the sample that are others | | | | |  | | | | | |
|  | | Mean number of chronic illnesses | | | | | For studies focusing on medications for a single medical condition, code 1 only if the study specifically states that the subjects have no medication condition other than the one for which the medications under study are being administered. | | | | | |
|  | | SD for number of chronic illnesses | | | | |  | | | | | |
|  | | Duration of T2DM in year mean | | | | | Average years being diagnosed T2DM | | | | | |
|  | | Insulin use: yes or no | | | | |  | | | | | |
|  | | Intervention group completion rate | | | | | % subject completed study from intervention group: # completer at end /# participants at beginning in intervention group | | | | | |
|  | | Control group completion rate | | | | | % subject completed study from control group: # completer at end /# participants at beginning in control group | | | | | |
|  | | **Theory**  0 = unable to determine  1 = actual or originated from empowerment  2 = social cognitive theory | | | | | Transtheoretical Model (TTM) | | | | | |
|  | | **Concepts**  0 = unable to determine  1 = self-efficacy | | | | |  | | | | | |
|  | | **Behavioral Intervention Components Items** Action plan, assessment, feedback, goal identification and setting, knowledge and skill enhancement, self-monitoring, motivational interviewing or MI, problem-solving, efficacy enhancing, role play, role modeling or peer support, SM support, promote collaboration/interaction with providers, reinforcement, empowerment, | | | | | The categories are not mutually exclusive. Some intervention strategies may be coded in more than one category. | | | | | |
|  | | Diet intervention  0 = no for items not mentioned  1 = yes | | | | |  | | | | | |
|  | | physical activity intervention  0 = no for items not mentioned  1 = yes | | | | |  | | | | | |
|  | | Complication management | | | | |  | | | | | |
| **Intervention Delivery Characteristics** | | | | | | | | | | | | |
|  | | Intervention individually tailored  0 = no  1 = yes | | | Tailoring requires that the intervention is customized to match selected characteristics of individual subjects (as compared to intervention that is the same for all subjects or interventions that are the same for subgroups of subjects [targeted intervention]). | | | | | | | |
|  | | Targeted intervention  0 = no  1 = yes | | | Targeted interventions are designed specifically for subgroups within the larger sample, but not for individuals (tailored). Thus an intervention that contains different content for male vs. female subjects or for people on hypertension drugs vs. those on diabetes drugs would be targeted. | | | | | | | |
|  | | Intervention delivery setting:  0 = unable to determine/ not applicable  1 = ambulatory care facility  2 = in-patient facility/hospital  3 = subjects’ homes  4 = religious/spiritual center/church  5 = community center other than health care facility or religious center  6 = pharmacy  7 = combination of settings | | | - Code for where the intervention is delivered. Code as subjects’ homes if the intervention is entirely mediated (e.g., by phone or mail). - If subjects receive something while in clinic they are supposed to implement at home, code the intervention delivery site as ambulatory care facility. - For interventions that occurred in ambulatory care clinics or pharmacies that are located within hospitals, code clinic or pharmacy as appropriate, NOT hospital. | | | | | | | |
| **Interventionist profession “**Interventionist” is defined as a person who delivers the intervention directly to patients or subjects.  0 = profession not mentioned 1 = yes, this profession mentioned | | | | | | | | | | | | |
|  | | Interventionist  1= an RN not advanced practice nurse (APN)  2 = an advanced practice nurse (APN)  3 = a nurse, credentials unspecified  4= a physician  5= pharmacist  6= PT  7=OT  8= clinical psychologist and nurse  9 = registered dietitian  10 = RN and RD | | | | | | | | Not clinical nurse specialist (CNS) or nurse practitioner (NP)  Could be described as clinical nurse specialist (CNS), nurse practitioner (NP), nurse midwife (CNM), or nurse anesthetist (CNA). | | |
| **Intervention Dose & Format: Code for interventions involving patients/subject contact, NOT train the trainer interventions** | | | | | | | | | | | | |
|  | | Number of intervention sessions | | | | - This does NOT include contacts used for data collection, unless the contact included both data collection and intervention. - If it is unclear which sessions are devoted to MA or if only portions of each session were devoted to MA, then count ALL sessions. - Mailed interventions would be counted as 1 time for every mailing. - If subjects receive a packet of educational materials and are instructed to read one piece of the packet at different times, count the number of sessions as one since they received all the material at the same time. | | | | | | |
|  | | Number of minutes for each intervention session | | | | - If a mean is reported, code the mean. - If a range is reported, code the midpoint value unless report information suggests otherwise—for example, do not code a middle value if 90% of intervention contacts are 2 hours long and the other 10% are only 5 minutes long—in this case, code 2 hours. - If the numbers of minutes vary – calculate a mean if feasible. - If the duration of some intervention sessions is reported, but duration of other sessions isn’t, code DASH. - Code ALL mailed interventions as a DASH (includes audio/visual materials in which recorded duration is reported). - Code telephone interventions for the number of minutes if this information is provided. | | | | | | |
|  | | Frequency of intervention  0 = unable to determine  1= weekly  2 = monthly  3 = weekly and monthly  4 = weekly, biweekly and monthly | | | |  | | | | | | |
|  | | Length of intervention in days | | | | - Code the days from the beginning of any intervention until the interventionists no longer had any contact with subjects (except exclusively for data collection). - To convert months to day, use 30.1 as the multiplier | | | | | | |
|  | | Delivery mode  1 =Telephone/cell phone  2 = telehealth system with visual + auditory transmission  3= Web/Internet/Email/text message  4 = computer but not Web/Internet/Email  5= written materials exclusively used  6= Face-to-face  7= mail  8 = mobile app | | | | 1. Code yes if any of the intervention was delivered through the telephone. Telephone includes live conversations or voicemails.   If the intervention is delivered primarily by means other than phone calls, but phone calls can be made at the interventionist’s discretion (“if needed” phone calls) and such calls are clearly part of the study protocol, code yes.   1. Any of the intervention was delivered through a system that allowed live visual+audio linkup between subjects and investigators/health care providers. This includes systems like telehealth, Skype, Webcams. 2. intervention was delivered through the Internet, Web or text message. 3. any of the intervention was delivered through a computer such as a software program at an ambulatory care setting. 4. the intervention was EXCLUSIVELY written materials that were given to the Ss. 5. any of the intervention was delivered face-to-face. 6. intervention was delivered through the mail. | | | | | | |
|  | | Tx format (social setting)  0 = unable to determine  1= single patient only  2 = patient + support system (e.g., family member or friends)  3 = group of patients | | | |  | | | | | | |
|  | | Tx recipient  0 = unable to determine  1= patient  2 = patients’ support system and/or caregivers  3 = patient’s care providers | | | |  | | | | | | |
| **Outcome Measures & Effect Size Data** | | | | | | | | | | | |  |
|  | | Post Treatment measurement timing  1^st^ day of data collection after Tx | | - Actual number of days between intervention and measurement for variable. - Record the number of **days** between the completion of the intervention and 1^st^ data collection after intervention. | | | | | | | | |
|  | | Length of follow up in days  Last day of data collection after Tx | | - Days between the completion of the first measure and the last measure (e.g, first measure is at baseline , and the last measure was completed at 6 months, so length of FU = 6 x 30 = 180 | | | | | | | | |
|  | | Time points of measurement | | - Number of measure points (e.g. baseline and 6 months = 2, baseline and every 3 months for 9 months = 4 every 3 months for 1 year) | | | | | | | | |
|  | | Outcomes of study | | - Include both primary and secondary outcomes | | | | | | | | |
|  | | Measure instruments  0 = no  1=list the instrument for each measure | | - List the name of measure instruments for each outcome | | | | | | | | |
| **Pre-intervention (baseline) Treatment Group A1C** | | | | | | | | | | | | |
|  | | Number of treatment subjects for which baseline data were calculated | | | | | | |  | | | |
|  | | Treatment group baseline mean A1C | | | | | | | Use absolute means. Do not use adjusted means. | | | |
|  | | Treatment group baseline A1C SD | | | | | | | Data in parentheses following means could be either SD or SE. Check table headers/footers for this information, or the analysis section of the report. | | | |
|  | | Treatment group baseline A1C SE | | | | | | |  | | | |
| **Pre-intervention (baseline) Control Group A1C** | | | | | | | | | | | | |
|  | | Number of Control Subjects for which baseline data were calculated | | | | | | |  | | | |
|  | | Control group baseline mean A1C | | | | | | |  | | | |
|  | | Control group baseline A1C SD | | | | | | |  | | | |
|  | | Control group baseline A1C SE | | | | | | |  | | | |
|  | | Difference between Tx and control group at baseline  0 =unable to determine, no data report  1= no difference  2= significant different | | | | | | |  | | | |
| **Post-intervention Treatment Group A1C** | | | | | | | | | | | | |
|  | | Number of Treatment Subjects in time intervention completed for Tx group | | | | | | |  | | | |
|  | | Treatment group outcome mean | | | | | | |  | | | |
|  | | Treatment group outcome SD | | | | | | |  | | | |
|  | | Treatment group outcome SE | | | | | | |  | | | |
| **Post-intervention Control Group A1C** | | | | | | | | | | | | |
|  | | Number of Control Subjects in time intervention completed for Tx group | | | | | | |  | | | |
|  | | Control group outcome mean | | | | | | |  | | | |
|  | | Control group outcome SD | | | | | | |  | | | |
|  | | Control group outcome SE | | | | | | |  | | | |
| **Direction of Effect Items** | | | | | | | | |  | | | |
|  | | Effect in the hypothesized direction for Treatment vs. Control?  1 = treatment group outcome better than control group  0 = treatment and control group outcome are the same  -1 = control group outcome is better than the treatment group | | | | | | |  | | | |
| **T statistic values** | | | | | | | | | | | | |
|  | | t value – post-test comparison between treatment and control groups; may be called ‘independent’ t-test | | | | | | | | | Be sure to use the t value that compares the means of the Treatment versus the Control groups. | |
|  | | P value associated with two group independent t-test | | | | | | | | | Do not use a cutoff value for statistical significance in this space. | |
| **Success rates** | | | | | | | |  | | | | |
|  | | Significant change of targeted concept(s)  0 = not mentioned, unable to determine  1= yes  2= no change | | | | | |  | | | | |
|  | | % subjects with A1C range 6-7 in treatment group pre-intervention (baseline) | | | | | | % subjects with A1C range 6-7 as proportion in treatment group at baseline.  $\frac{number of subjects in treatment group with A1C btw 6 and 7}{total number of subjects in treatment group at baseline}$ | | | | |
|  | | % subjects with A1C range 6-7 in control group pre-intervention (baseline) | | | | | | % subjects with A1C range 6-7 as proportion in control group at baseline.  $\frac{number of subjects in control group with A1C btw 6 and 7}{total number of subjects in control group at baseline}$ | | | | |
|  | | % subjects with A1C range 6-7 in treatment group post-intervention | | | | | | % subjects with A1C range 6-7 as proportion in treatment group after intervention.  $\frac{number of subjects in treatment group with A1C btw 6 and 7}{total number of subjects in treatment group after intervention}$ | | | | |
|  | | % subjects with A1C range 6-7 in control group post-intervention | | | | | | % subjects with A1C range 6-7 as proportion in control group after intervention.  $\frac{number of subjects in control group with A1C btw 6 and 7}{total number of subjects in control group after intervention}$ | | | | |
|  | | Intervention subjects post intervention FBS mean | | | | | |  | | | | |
|  | | Intervention subjects post intervention FBS SD | | | | | |  | | | | |
|  | | Control subjects post intervention FBS mean | | | | | |  | | | | |
|  | | Control subjects post intervention FBS SD | | | | | |  | | | | |
|  | | T value: post-test comparison between treatment and control groups; may be called ‘independent’ t-test. | | | | | | Be sure to use the t value that compares the means of the Treatment versus the Control groups. | | | | |
|  | | P value associated with two group independent t-test | | | | | | Do not use a cutoff value for statistical significance in this space. | | | | |
|  | | procedures manage missing data  0 = no for items not mentioned  1 = yes | | | | | |  | | | | |
|  | | Reliability and validity of instruments reported  0 = no for items not mentioned  1 = yes | | | | | |  | | | | |
|  | | Statistical software used  0 = no for items not mentioned  1 = yes | | | | | |  | | | | |
|  | | Sample size estimate (power analysis) | | | | | |  | | | | |
|  | | Intention-to-treat analysis | | | | | |  | | | | |
|  | | Flow diagram | | | | | |  | | | | |
|  | | Baseline table | | | | | |  | | | | |
|  | | Paper quality score | | | | | |  | | | | |
|  | | LOE | | | | | |  | | | | |
