# Supplementary material for: Effectiveness of Social Cognitive Theory–Based Interventions for Glycemic Control in Adults With Type 2 Diabetes Mellitus: Protocol for a Systematic Review and Meta-Analysis
Source: JMIR Res Protoc. 2020 Sep 2;9(9):e17148. doi: 10.2196/17148 (PMC7495254; doi:10.2196/17148)
Supplement: Multimedia Appendix 4 [file resprot_v9i9e17148_app4.docx]

| **SCT Concept** | **Description** |
| --- | --- |
| Social learning | Acquired behavior from observing or imitating others. |
| Social cognition | Knowing, reasoning, perceiving, or mental judgment. |
| Feedback | Decision-making guidance through dialogue. |
| Knowledge | Gained understanding or skill through educations or ecperience. |
| Attitude | A way of thinking that forms behavior. |
| Self-efficacy | A perception of personal control or of ability to accomplishing self-management activities. |
| Efficacy enhancement | Positive perception of self-management ability. |
| Coping | Behavioral or cognitive ability to manage circumstances perceived as stressful. |
| Goal setting | Short and long-term goals individualized and targeted to individual needs |
| Self-regulation | The aptitude of an individual to self-manage, control, and evaluate behavior. |
| Belief | Acceptance or certainty of something. |
| Mastery | Demonstration of knowledge or a skill. |
| Arousal | A self-preserving behavior brings about anxiety leading to an avoidance of behaviors. |
| Efficacy expectation | A belief that an outcome can be accomplished. |
| Problem solving | A mechanism to cope with a challenge. |
| Accomplishment | Require knowledge and skill acquisition, uniting and reinforcing cognitive processes. |
| Verbal persuasion/motivation | Pursuasian to respond in the face of reward or punishment. |
| Vicarious experience | Delivers an experience to another person |
| Social support, community, social norms,  experience | Informational support e.g., education or advice. Instrumental support e.g. financial support or physical assistance with self-management actions. Emotional support e.g., acceptance and approval. Affirmational support e.g., validation of self-management efforts |
| Reward | Recognition or achievement. |
| Physiological or effective state | Physical or emotional domains for receiving information or an experience. |
| Reflection | A self-regulating learning method |
