# Supplementary material for: Effectiveness of Social Cognitive Theory–Based Interventions for Glycemic Control in Adults With Type 2 Diabetes Mellitus: Protocol for a Systematic Review and Meta-Analysis
Source: JMIR Res Protoc. 2020 Sep 2;9(9):e17148. doi: 10.2196/17148 (PMC7495254; doi:10.2196/17148)
Supplement: Multimedia Appendix 5 [file resprot_v9i9e17148_app5.docx]

|  | Feedback | Knowledge | Self-efficacy | Self-management | Self-monitoring | Self-regulation |
| --- | --- | --- | --- | --- | --- | --- |
| 16_Rosal_Diabetes.txt | 2 | 6 | 4 | 8 | 7 | 1 |
| 44_Ha_Effects.txt | 1 | 15 | 41 | 7 | 2 | 0 |
| 49_Cai_Effectiveness.txt | 8 | 16 | 18 | 14 | 1 | 0 |
| 77_Sinclair_Outcomes.txt | 0 | 4 | 1 | 19 | 0 | 0 |
