# Supplementary material for: Effectiveness of Social Cognitive Theory–Based Interventions for Glycemic Control in Adults With Type 2 Diabetes Mellitus: Protocol for a Systematic Review and Meta-Analysis
Source: JMIR Res Protoc. 2020 Sep 2;9(9):e17148. doi: 10.2196/17148 (PMC7495254; doi:10.2196/17148)
Supplement: Multimedia Appendix 7 [file resprot_v9i9e17148_app7.docx]

| **Domain** | **Support for judgment** | **Review authors’ judgment** |
| --- | --- | --- |
| ***Selection bias*** |  |  |
| **Random sequence generation** | Describe the method used to generate the allocation sequence in sufficient detail to allow an assessment of whether it should produce comparable groups. | Selection bias (biased allocation to interventions) due to inadequate generation of a randomized sequence. |
| **Allocation concealment** | Describe the method used to conceal the allocation sequence in sufficient detail to determine whether intervention allocations could have been foreseen in advance of, or during, enrolment. | Selection bias (biased allocation to interventions) due to inadequate concealment of allocations prior to assignment. |
| ***Performance bias*** |  |  |
| **Blinding of participants and personnel *Assessments should be made for each main outcome (or class of outcomes)*** | Describe all measures used, if any, to blind study participants and personnel from knowledge of which intervention a participant received. Provide any information relating to whether the intended blinding was effective. | Performance bias due to the knowledge of the allocated interventions by participants and personnel during the study. |
| ***Detection bias*** |  |  |
| **Blinding of outcome assessment *Assessments should be made for each main outcome (or class of outcomes)*** | Describe all measures used, if any, to blind outcome assessors from knowledge of which intervention a participant received. Provide any information relating to whether the intended blinding was effective. | Detection bias due to the knowledge of the allocated interventions by outcome assessors. |
| ***Attrition bias*** |  |  |
| **Incomplete outcome data *Assessments should be made for each main outcome (or class of outcomes)*** | Describe the completeness of outcome data for each main outcome, including attrition and exclusions from the analysis. State whether attrition and exclusions were reported, the numbers in each intervention group (compared with total randomized participants), reasons for attrition/exclusions where reported, and any re-inclusions in analyses performed by the review authors. | Attrition bias due to amount, nature or handling of incomplete outcome data. |
| ***Reporting bias*** |  |  |
| **Selective reporting** | State how the possibility of selective outcome reporting was examined by the review authors, and what was found. | Reporting bias due to selective outcome reporting. |
| ***Other bias*** |  |  |
| **Other sources of bias** | State any important concerns about bias not addressed in the other domains in the tool.  If particular questions/entries were pre-specified in the review’s protocol, responses should be provided for each question/entry. | Bias due to problems not covered elsewhere in the table. |
