# Supplementary material for: Effectiveness of Social Cognitive Theory–Based Interventions for Glycemic Control in Adults With Type 2 Diabetes Mellitus: Protocol for a Systematic Review and Meta-Analysis
Source: JMIR Res Protoc. 2020 Sep 2;9(9):e17148. doi: 10.2196/17148 (PMC7495254; doi:10.2196/17148)
Supplement: Multimedia Appendix 8 [file resprot_v9i9e17148_app8.docx]

| Study | Topic | Result |
| --- | --- | --- |
| Author, year | Publication type  Method  Participants  Treatment/control  Mean age  Gender  Race  White  Black  Hispanic  Other  Mean duration of T2DM  Intervention Theory  Intervention format  Control  Intervention duration  Outcomes  Follow up  Notes | E.g., Peer-reviewed journal  Randomized control trial  N=50  25/25  58  50% female  75%  22%  3%  0  5  SCT  Role modeling, knowledge enhancement, reflection  Routine care  3 months  HgBA1c, self-efficacy  12 months |
